# Spontaneous Condyle-Like Development after Total Resection of Mandible Giant Osteochondroma: Case Report and a Follow-Up for Five Years

**DOI:** 10.1155/2020/3720909

**Published:** 2020-02-06

**Authors:** Marco Antonio de Oliveira Filho, Luis Eduardo Almeida, Andrea Duarte Doetzer, Allan Fernando Giovanini, Osvaldo Malafaia

**Affiliations:** ^1^Evangelic University Hospital, Alameda Augusto Stellfeld, 1908 Curitiba, Brazil; ^2^Marquette University, 1801 W. Wisconsin Avenue, Milwaukee, USA; ^3^Pontifícia Universidade Católica do Paraná, Rua Imaculada Conceição, 1155 Curitiba, Brazil

## Abstract

Osteochondroma manifests as a benign tumor that occurs as an abnormal bony development. This tumor is commonly asymptomatic and presents an exophytic outgrowth on bone surfaces, near synovial joints, a condition that invariably induces evident facial deformities. Treatment for this type of tumor usually involves a surgical approach promoting a total or partial resection of the affected anatomical area associated to prosthetic reconstruction of the bone area extracted. We present a case report about a giant mandibular condyle osteochondroma in a 37-year-old female patient. Her treatment involved a total condylectomy without immediate condylar reconstruction, which would be performed in a posterior surgical approach. During the patient's follow-up (every 6 months of post operation), a spontaneous and rudimentary condyle-like formation was observed. Because the stomatognathic function and facial harmony were satisfactory, we observed the condyle-like development for 5 years of follow-up. Also, because both the aesthetic aspect and functional evolution of the maxillary bone were considered satisfactory, no complementary reconstruction surgical treatment was required for the giant osteochondroma of the mandibular condyle.

## 1. Introduction

Osteochondroma (OC), also known as osteocartilaginous exostosis, constitutes a benign bone neoplasm originating from the bone surface. In its microscopy aspect, OC is invariably composed of cortical and/or trabecular bone and surrounded by evident hyaline cartilage [1, 2].

The OC is uncommon in the head and neck areas, but, when present in the craniomaxillary topography, manifests as a painless exophytic mass that grows slowly and predominately affects the mandibular condyle [3], followed by the mandibular coronoid process [4]. This condition commonly results in temporomandibular joint dysfunction, facial deformity, and dental malocclusion [5].

The literature emphasizes that condylar OC should be treated with surgical treatments, which include resection through a conservative condylectomy, total condylectomy with posterior reconstruction, or selected tumor removal without condylectomy when the tumor is considered small. However, it should be noted that a conservative resection may preserve a part of the mandibular head but the recurrence index of the tumor is higher. On the other hand, a total condylectomy demands condylar replacement with a costochondral graft and preservation of the articular disc. This approach improves morbidity and also may result in loss of vertical dimension, occlusal interference, and mandibular deviation during mouth opening [4].

We present a case report about a giant mandibular condyle OC that was treated with total condylectomy without immediate condylar reconstruction. Besides its significant size, an uncommon peculiarity of this case is the spontaneous regenerative-like process mimicking a rudimentary “new condyle” formation, which improved the patients' aesthetics and mandibular functional movements.

## 2. Presentation of Case

**Figure 1:**
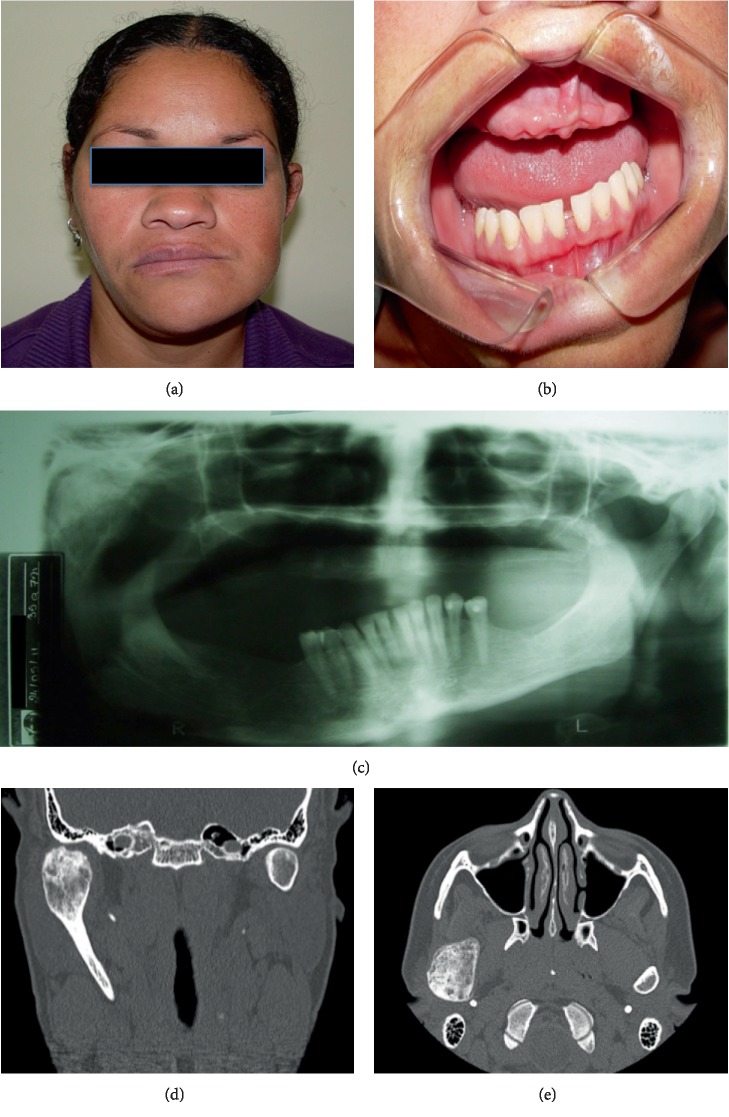
(a) Clinical facial aspect before surgery. The patient presented facial elongation, with a left chin deviation. (b) Occlusal plane alteration of the mandible. Patient with an edentulous maxilla. (c) Panoramic RX showing a tumoral mass associated to the right mandibular condyle. (d, e) Coronal and axial CT planes showing the size and limits of the tumor.

A 37-year-old female patient was referred for professional evaluation at the Maxillofacial Surgery Department of Evangelical-Mackenzie University Hospital of Curitiba, Brazil. She complained of slow and progressive facial asymmetry over an evolution of 6 years. During clinical examination, a dentofacial deformity with the chin deviating to the left side was detected, along with an elongation of the right mandibular side and asymmetry of the occlusal plane (Figures 1(a) and 1(b)). In an oral inspection, a superior and inferior edentulism was evident, but the patient had a normal mouth opening. She previously possessed a panoramic X-ray (Figure 1(c)) but underwent a computed tomography (CT), from which a sessile mass with large volume attached to the right mandibular condyle was observed (Figures 1(d) and 1(e)).

**Figure 2:**
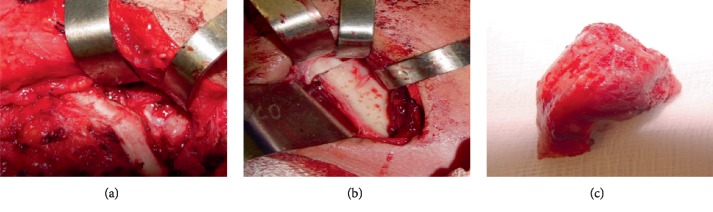
(a) Retromandibular access. (b) Hemicoronal access. (c) Osteotomy line mass removed with little safety margin.

Based on the clinical and tomographic aspects, surgical excision with total condylectomy was the chosen for treatment. Due to the large dimensions of the tumor, hemicoronal access with preauricular extension and retromandibular incision was performed. The retromandibular access allowed the osteotomy to be performed, and the hemicoronal access enabled the lesion to be released (Figures 2(a)–2(c)). The masseter muscle was deinserted from its original place and reinserted through sutures to the zygomatic arch. The suture was done for each plane separately, then the suction drain was installed. The lesion was removed and analyzed histopathologically, and then the diagnosis of OC was established. The patient was followed up for 5 years, and every 6 months, a CT was taken to evaluate the postsurgical site. This patient presented no postsurgical complications.

**Figure 3:**
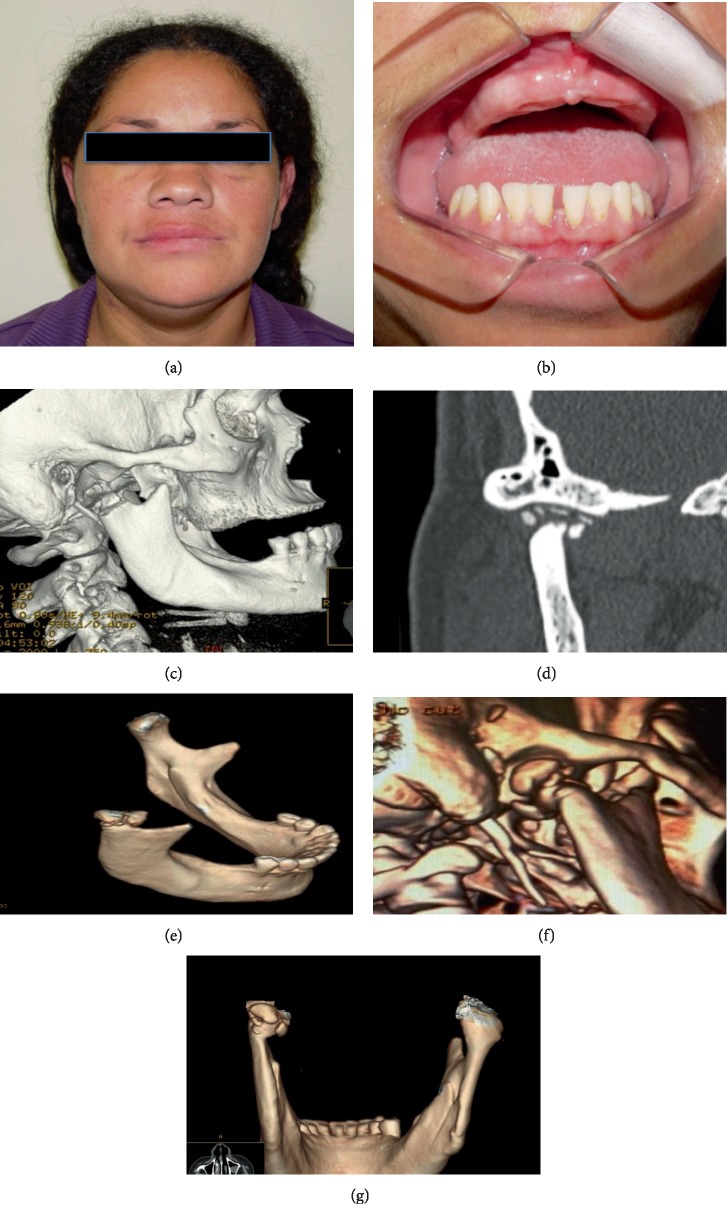
(a) Clinical aspect after surgery, with a slight improvement in facial asymmetry. (b) Mandible occlusal plane correction. (c) CT post resection image, without immediate reconstruction. (d) Tomography revealing the “islands of ossification” (arrows) 24 months of post surgery. (e, f) The confluence of ossification islands, with a “new condyle” formation after 3 years (arrows). (g) Image of a 5-year follow-up. The “new condyle” with a shape closer to a normal condyle (arrow).

Follow-up CT images taken 24 months post operation showed islands of ossification localized in the space between the osteotomy and mandibular cavity of the temporal bone (Figure 3(d)). At this moment, the patient presented no facial asymmetry (Figures 3(a) and 3(b)). Moreover, she reports no pain and all mandibular movements have been preserved. Therefore, we decided to evaluate the patient periodically instead of submitting her to an exploratory surgery. After 3 years, a confluence of the islands of ossification in tomography was verified (Figures 3(e) and 3(f)), with a consequent spontaneous and rudimentary condyle formation. The control images taken after 5 years showed a remodeling of the “new condyle,” toward the shape of an intact condyle (Figure 3(g)). The patient remained without pain, with all mandibular movements preserved and without perceptive volume alteration in this region.

## 3. Discussion

An OC constitutes a benign tumor that usually evolves slowly and asymptomatically until it reaches excessive proportions. Its etiology remains unclear, but there is a likely theory that also strictly correlates to the hypothesis of an aberrant epiphyseal cartilage present in the cortical bone [5]. Stress at the insertion point of the tendons where there is an accumulation of potentially cartilaginous cells could be a contributing factor to the OC's formation [6]. This fact could explain the higher incidence of OC affecting the anteromedial mandibular condyle (the insertion of the pterygoid lateral muscle), when compared to other sites of craniofacial topography.

Regarding treatment of condylar OC, an important issue that should be considered is if a condylar reconstruction should be, in fact, performed after total condylectomy [4].

It is no doubt that approach for impeccable positioning of the mandibular maxillary complex is sometimes critical, especially in craniofacial deformity cases, where occurs asymmetry associated to maxillary horizontal plane deflect, midline anomaly, and deviation of the inclination of the teeth. In these severe cases, there is an evident benefit of 3D surgical planning using surgical guide for prosthetic reconstruction in order to improve facial harmony [7].

Thus, the use of 3D design and waferless combined is becoming a common protocol in orthognathic surgery. According to literature, there is a main benefit of waferless maxillary positioning which is the very high accuracy transferring the preoperative plan into reality [8]. According to Heufelder et al. [9], this perspective is based on location of the screws that they are all indicated through of a surgical guide condition that facilitates the surgeon's handling, since the surgeon can drill all the screw holes at once and posteriorly insert all the screws when the final position is confirmed. Besides that, this technique does not demand any intermaxillary fixation and wire manipulation for maxillary positioning, a condition that also favors the postoperative period.

Agreeing with this perspective, Wolford et al. [10] reviewed 37 patients with OC and noted success in cases treated with exeresis of the whole condyle with orthognatic surgery. However, González-Otero et al. [11] reviewed previously published articles and noted that several cases of condylar OC were excised with condylectomy without immediate reconstruction. Based on their observations, due to several clinical postoperative complications, the authors advised a surgical approach to avoid secondary deformity from vertical shortening on the lesion side.

In the present case report, due to the patient's large tumor size, a resection of the lesion with posterior customized prosthesis reconstruction was planned. It was a surprise that the initial results were satisfactory. The patient presented acceptable mouth opening and adequate stomatognathic function.

Thus, due to satisfactory postoperative facial harmony and high costs of technical waferless associated with orthognathic surgery protocol, the patient declined an immediate reconstructive procedure because her results were above expectations. Thus, we performed a postsurgical follow-up semiannually for 5 years. The CT images taken for our group demonstrated images that suggested a “new condyle-like formation.”

In fact, it is likely to induce a new condyle formation after condylar fracture by functional treatment in growing patients, but it is unusual in patients with postoperative final bone development. However, similar to our results, some authors reported spontaneous bone regeneration in adults following resection of some proportions of the mandible. To explain these results, Fell [12] showed that periosteal cells may survive following surgery and may remain in the surgical bed and exhibit osteogenic potential.

Besides that, a hypothesis that should be considered for capacity of the rudimentary condyle neoformation is a likely hyperplasia of a residual tumor-free mandibular condyle. In areas with a condylar surgical bed, there is usually a greater amount of cancellous bone when compared to compact bone. It is noteworthy that the medullar bone, combined simultaneously with fluids from the remaining joint capsule and disc soft tissues fragments, could provide a microenvironment where hyperplasia of mesenchymal stem cells present in reminiscent cancellous bone would occur, resulting in neochondrogenesis and posterior osteogenesis [13]. This hypothesis may be inferred since there was an evident rudimentary condylar structure with no clinical comorbidity or ankylosis.

We followed up on a patient who underwent total osteocondylectomy to remove an OC without further condylar reconstruction. In this specific and unusual case report, a condyle-like structure was formed, not only restoring stomatognathic functions but also restoring the facial harmony of the patient.

However, despite the success achieved so far in this patient follow-up, it should be highlighted that the case presented here is unique, and except for the operating costs, the real benefits of the technique presented here with the established techniques using surgical guides remain an inference, since more cases are needed for efficient comparison. However, the case presented here may give a new treatment alternative for low-income people who cannot afford the high costs of the conventional protocol.
